# Soft Tissue Augmentation with Autologous Fat Graft: The Dissected Pouch Technique

**DOI:** 10.4103/0974-2077.53095

**Published:** 2009

**Authors:** Murat Livaoğlu, Ercan Yavuz

**Affiliations:** *Department of Plastic and Reconstructive Surgery, Karadeniz Technical University, School of Medicine, Trabzon, Turkey*; 1 *Department of Plastic and Reconstructive Surgery, Fatih State Hospital, Trabzon, Turkey*

**Keywords:** Autologous fat graft, dissected pouch, lipofilling, soft tissue augmentation

## Abstract

**Background:**

Soft tissue augmentation with autologous fat graft has been increasingly used by plastic surgeons despite unpredictable results. Several techniques have been described to prevent the main setback of this technique, fat graft resorption. However, there is no ideal technique described for this purpose.

**Materials and Methods:**

Twenty patients with subcutaneous tissue loss, atrophy or hypoplasia were treated with lipofilling. A subcutaneous pouch is dissected at the deformed area and later it is filled with autologous fat graft.

**Results:**

Graft maintenance during the postoperative period was satisfactory. Overcorrection was not performed except for the first three cases. Patient, surgeon and layman satisfaction was surveyed. Satisfaction was rated between 0 and 10. The mean score was 7.67 ± 1.22.

**Conclusion:**

The authors describe a technique for soft tissue augmentation which effectively corrects contour deformities, provides a low resorption rate and a relatively non-visible scar without causing irregularities.

## INTRODUCTION

Despite unpredictable results, clinical use of autologous fat transfer for soft tissue augmentation has increased among plastic surgeons. The rising interest in this procedure has paralleled the development and popularity of liposuction for body contouring This popularity may be due to the opportunity of having an already present material at hand to augment or restore areas of the face with volume loss or contour irregularities. Additionally currently available filler materials such as hyaluronic acid, bovine collagen, polymethyl methacrylate, calcium hydroxyapatite suffer from distinct disadvantages.

While satisfactory results can be achieved in the early postoperative period, long-term results may be disappointing for both the patient and the surgeon. The most common difficulty is the estimation of the resorption rate. Several techniques have been suggested for maximum fat survival including atraumatic fat harvesting technique, centrifugation, graft washing and addition of growth factor. However, there is no ideal technique described for this purpose.

The authors of this paper describe an alternative technique for soft tissue augmentation: Placement of autologous fat graft in a dissected pouch. A smooth and regular surface could be obtained with this soft tissue augmentation technique. The intervention also results in a relatively invisible scar.

## MATERIALS AND METHODS

From August 2005 to July 2007, 20 patients (15 females and 5 males, age range 23-56 years) with subcutaneous tissue loss, atrophy or hypoplasia were treated with the described technique in our clinics. Patient characteristics, augmented area, incision localizations, filled volume (ml) are summarized in Table 1. The patients were informed about the technique and alternative options including their benefits, limitations, and complications.

**Table 1:** Demographic profile and operative data of study patients

Age/ Sex	Augmented area	Incision	Volume ml
32/F	Bilateral, trochanter	Gluteal crisis	2×85
34/F	Multiple depression areas on the left thigh	Multiple incision	2-3 per incision
35/F	Left zygomatic depression	Hairline	3
26/M	Bilateral cheek	Preauricular	2×11
42/F	Bilateral cheek	Preauricular	2×7
56/F	Bilateral cheek	Preauricular	2×7
23/F	Bilateral malar augmentation	Preauricular	2×7
33/F	Bilateral medial thigh	Medial knee crisis	2×95
46/F	Left cheek (postradiotherapy)	Nasolabial	26
24/M	Right malar depression	Nasolabial	8
27/F	Bilateral facial atrophy	Preauricular	2×45
22/F	Right anterior thigh	Old scar	8
24/F	Left hemifacial atrophy	Nasolabial	11
30/F	Left hemifacial atrophy	Preauricular	14
53/M	Post-maxillectomy depression	Nasolabial	33
26/M	Glabellar depression scar	Old scar	4
27/M	Right supraorbital depression scar	Old scar	5
27/F	Bilateral malar augmentation	Nasolabial	2×5
32/F	Bilateral malar augmentation	Nasolabial	2×7
30/F	Right lateral thigh	Old scar	50

### Surgical technique

Areas to be augmented were marked. Using deep sedation, the recipient area and lower abdomen or medial thigh were prepared with povidone iodine and draped. The tumescent solution containing 0.08% lidocaine and 1:500,000 of epinephrine was infiltrated into the donor and recipient sites. Approximately 0.5-1 cm skin incisions were made in the appropriate place. For example, preauricular incision was preferred for hemifacial augmentation and nasolabial fold incision for malar augmentation. Subcutaneous dissections were performed with blunt scissors and extended 1-2 cm further to the marked area. The dissection plane was between dermis and local fat tissue. After dissection was completed, gentle compression was applied while the fat graft was being harvested.

Fat graft was harvested using a 5-mm blunt, three-holed suction cannula, attached to a 50-ml syringe. Preferred donor sites were the lower abdomen in 13 patients, and lower extremity in seven patients. After insertion of the cannula, under mild negative pressure, fat graft was aspirated with the aid of repeated back and forth motions of cannula. Approximately 10-20 ml fat graft per syringe was harvested. By aspiration of saline, the fat graft within the syringe was washed off from blood, oil and tumescent solution. Harvested fat was injected into the dissected plane via incision and then incision was sutured with fine polypropylene. Overcorrection was not performed with the exception of the first three cases. Irregularities were arranged with a gentle massage. The grafted sites were immobilized for a week with a compression dressing. Antibiotic and anti-inflammatory medications were used in the postoperative period.

## RESULTS

**Figure 1:**
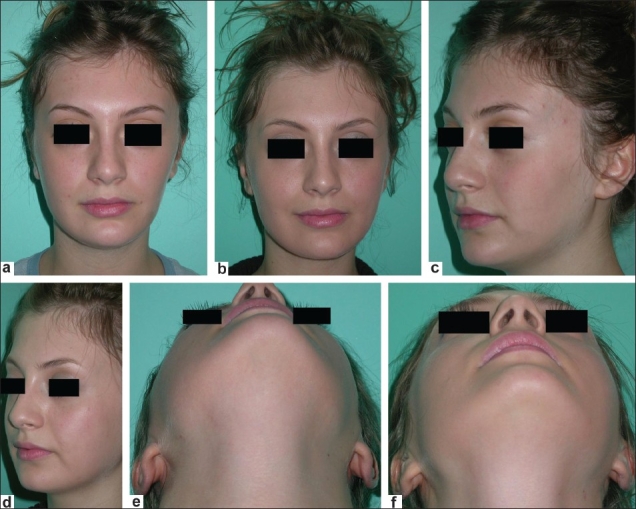
A 24-year-old woman with moderate left-sided hemifacial atrophy. Preoperative views (a,c,e), postoperative views (b, d, f). Nasolabial incision is unnoticeable

**Figure 2:**
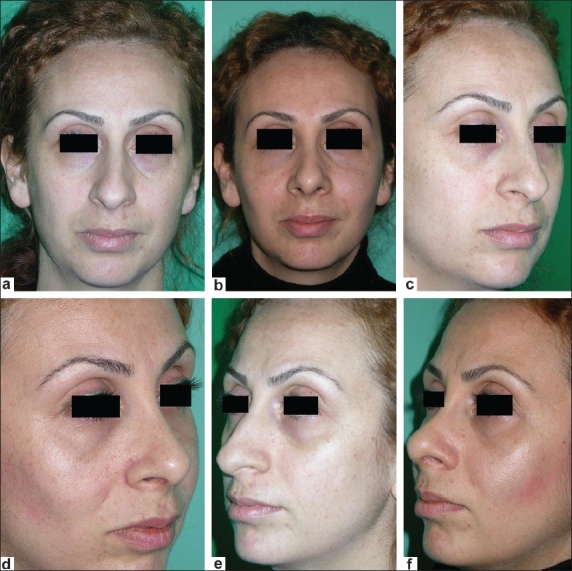
A 32-year-old woman who underwent bilateral malar augmentation. Preoperative views (a,c,e), postoperative views (b, d, f); 7 cc autologous fat graft per side was filled via nasolabial incision

**Figure 3:**
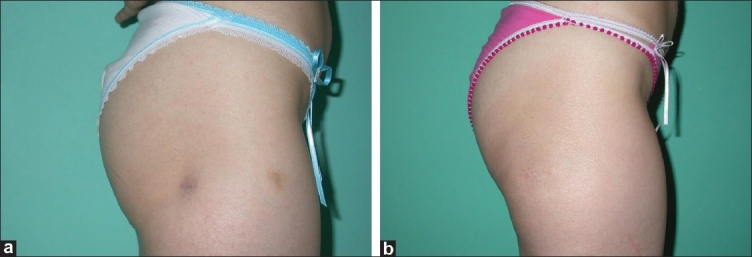
Right lateral thigh was dissected through old liposuction scar and then augmented with 50 cc fat graft

A total of 20 patients (facial augmentation in 15 patients, lower extremity intervention in five patients) underwent soft tissue augmentation with the described technique. Procedure-related complications were not observed in donor and recipient areas. In one patient, debulking was performed with liposuction at the tenth postoperative month due to persistence of overcorrection on left cheek. A new injection procedure was applied in one patient due to resorption. The mean postoperative follow-up was 1.7 years. The degree of improvement was scored by patients, surgeons and laymen. The satisfaction questionnaires were answered at the sixth postoperative month. Postoperative satisfaction was rated between 0 and 10, with 0 the worst and 10 the best grade. The overall satisfaction score was 7.67 ± 1.22 [Table 2]. Some results are shown in Figures 1–3.

**Table 2:** Satisfaction questionnaire

Patient	Surgeon	Layman	Overall
7.75 ± 1.02	7.56 ± 1.5	7.6 ± 1.14	7.67 ± 1.22

## DISCUSSION

Over the past two decades, treatment of soft tissue losses (congenital, acquired or senile) with lipofilling has shown encouraging clinical results in terms of texture, softness and quality of skin pattern. Autologous fat tissue has been considered as an ideal filler for soft tissue augmentation because it is biocompatible, versatile, stable, long-lasting, natural-appearing, readily available, abundant, inexpensive, and can be harvested easily and repeatedly, with minimal trauma to the donor sites.[1] Therefore autologous fat tissue transfers have been devised to reestablish volume and contour that have been lost due to various causes. Despite extensive clinical use and optimism, there remains an uncertainty among practitioners regarding viability of the transferred fat.

There are various autologous fat transfer techniques from the points of donor site, harvesting method, type of anesthesia and the injection technique for soft tissue augmentation. En bloc fat graft harvesting is the least traumatic technique to the graft and has been shown to reduce tissue loss and improve fat graft survival.[2] In a rabbit model investigating the effect of different techniques on fat graft survival, Marques *et al*., found that surgical excision increased survival rates. However, surgically excised fat tissue transfer results with both donor and recipient area scars. Even if the donor area is preferred at a non-visible localization or the graft is harvested using a previous scar, en bloc fat cannot be introduced from a small incision. Therefore, syringe aspiration of fat appears to be the most popular method for fat harvesting.[3,4]

High fat graft resorption rates have been attributed to traumatic handling of the graft during the harvest and the injection stages. A number of authors recommend the use of half of the normal suction pressure to avoid mechanical injury to adipocytes during aspiration.[5] The shaft of the needle may produce higher shear and wake vortices, resulting in decreased cell viability.[5] The use of a 5-mm diameter blunt suction cannula prevented traumatization of harvested fat tissue. With the wide-diameter cannula, macro particle fat graft could be obtained almost similar in size with en bloc graft.

The most important uncertainties of the use of aspirated fat graft injection are about the quantity of the graft and the required degree of overcorrection. Overcorrection is a vague concept and there is no agreement on the amount or even the need of it. Different resorption rates are described in the literature. According to our previous experience and the literature data, we made overcorrection in the initial three operations, like the other lipofilling techniques. Even after the end of the first postoperative year, we observed persistence of overcorrection. Moreover, we had to debulk the overcorrected fat in the patient. We have not performed overcorrection since this experience. We had to make an additional injection only in one patient after four months.

Some other uncertainties for autologous fat transfer are the appropriate fat graft injection level and whether to perform the injection multilaminar or not.[4] To improve survival of fat grafts after transplantation, several injection techniques, such as 3-4 mm pearl transfer, roll injection, multitunnel fan-shape injection and multilane radial injection have been suggested.[1,6–8]

The most common complication of autologous fat transfer is related to the placement of inappropriate amount of fat. Irregular resorption remains a problem when using autologous, nonvascularized free fat grafts for correction of soft-tissue defects. Growth factors, beta blockers, insulin, growth media, and hyperbaric oxygen have been used for the prevention of irregular resorption. Yet some authors recommend minimal handling and no preparatory treatment of the intended graft tissue.[5,9,10] The other common problem is the presence of irregularities during the injection. We did not observe any undesirable outcome except in the first three patients in whom overcorrection was performed. Remarkable irregularities were not observed in the recipient areas.

Fat injection through cannula with positive high pressure could be potentially destructive to the adipocytes' cellular membranes and ultimately reduce graft survival.[11] Excess volume injection leads to a positive pressure space for the fat graft. This may be another reason for reduced fat graft survival. Our fat graft technique eliminates trauma to the fat during the injection step of the procedure and also eliminates the pressure of the surrounding tissue. Unlike many fat injection techniques, this method enhances the potential for preservation of the adipocytes' cellular membranes and microcirculation, possibly resulting in an increased probability for successful survival of the fat graft.

In conclusion, our fat injection technique combines fat injection and en bloc technique, provides a low resorption rate, a relatively non-visible scar, which is an important problem for en bloc fat transfer and eliminates irregularities. Autologous fat transplantation with the described technique can effectively correct contour deformities that require moderate augmentation. We have had great success in our series utilizing dissected pouch filling.
